# Deep Eutectic Solvent Systems as Media for the Selective Extraction of Anti-Inflammatory Bioactive Agents

**DOI:** 10.3390/molecules30163357

**Published:** 2025-08-12

**Authors:** Beatriz Giner, Estela Sangüesa, Estefania Zuriaga, Laura Culleré, Laura Lomba

**Affiliations:** Facultad de Ciencias de la Salud, Universidad San Jorge, Campus Universitario, Autov. A23 km 299, Villanueva de Gállego, 50830 Zaragoza, Spain; bginer@usj.es (B.G.); esanguesa@usj.es (E.S.); ezuriaga@usj.es (E.Z.); lcullere@usj.es (L.C.)

**Keywords:** deep eutectic solvents, extraction, bioactive compounds, anti-inflammatory

## Abstract

Bioactive compounds (BCs) are naturally occurring molecules found in plants, fungi, and microorganisms that can provide health benefits beyond nutrition. However, in order to administer them, they must be extracted from these organisms. This study reviews the extraction of anti-inflammatory bioactive compounds using deep eutectic systems (DESs). It was found that DES extraction media can be categorized as either choline chloride-based or natural product-based (e.g., proline, betaine, and lactic acid). Results indicate that extraction yields depended on many factors such as extraction method and DES composition, with values ranging from 0.02 to 200 mg/g. For example, curcumin extraction using ChCl–propylene glycol (1:2), for example, reached 23.1 mg/g, whereas rutin extraction using ChCl–levulinic acid (1:2) achieved 200 mg/g. Regarding this, most of the eutectic mixtures used are choline chloride (ChCl)-based combined with sugars, polyalcohols, organic acids, or even water. Nonpolar DESs combining betaine, L-proline, amino acids, sugars, and organic acids have also been used for the extraction of BCs with anti-inflammatory potential. Although the use of DES offers significant advantages for extraction processes, certain limitations still need to be overcome. This review highlights the comparative advantages of DESs in terms of extraction efficiency and environmental sustainability, offering practical insights for selecting optimal systems to extract anti-inflammatory bioactive compounds.

## 1. Introduction

Inflammation is a fundamental biological process triggered by the immune system in response to infection, injury, or exposure to harmful agents. Its main functions are to eliminate the initial cause of cellular damage, clear necrotic cells and tissues, and initiate tissue repair. Acute inflammation is usually beneficial and self-limiting. It is characterized by a rapid onset and the involvement of immune cells, such as neutrophils. In contrast, chronic inflammation is a prolonged response that can persist for months or even years. It primarily involves macrophages and lymphocytes and is associated with ongoing tissue damage and repair [1]. Furthermore, chronic inflammation plays a significant role in the pathophysiology of various diseases, including rheumatoid arthritis, type 2 diabetes, cardiovascular disease, neurodegenerative disorders, and certain types of cancer. Persistent inflammatory stimuli, such as metabolic stress, gut dysbiosis, and environmental factors, can induce a low-grade, systemic inflammatory state that exacerbates disease progression and increases the risk of comorbidities [2].

Effective control of inflammation is essential to preventing systemic complications and irreversible tissue damage. Chronic inflammation not only impairs organ and system function but also contributes to the development of secondary conditions such as atherosclerosis and metabolic syndrome. Therefore, regulating inflammation is a key therapeutic goal in managing chronic diseases and improving patients’ quality of life [3].

### 1.1. Anti-Inflammatory Agents: Types and Mechanisms

Anti-inflammatory drugs modulate the inflammatory response by targeting specific molecular pathways. These drugs are classified into two main categories: non-steroidal anti-inflammatory drugs (NSAIDs) and corticosteroids. NSAIDs represent a diverse pharmacological class that exert their effects by inhibiting one of the two cyclooxygenase (COX) enzymes. These enzymes are involved in the arachidonic acid cascade, resulting in decreased prostaglandin (PG) synthesis. NSAIDs are generally grouped into several subclasses according to their chemical structure and selectivity: acetylated salicylates (e.g., aspirin); non-acetylated salicylates (e.g., diflunisal and salsalate); propionic acids (e.g., naproxen and ibuprofen); acetic acids (e.g., diclofenac and indomethacin); enolic acids (e.g., meloxicam and piroxicam); anthranilic acids (e.g., meclofenamate and mefenamic acid); naphthylalanines (e.g., nabumetone); and selective COX-2 inhibitors (e.g., celecoxib and etoricoxib) [4]. On the other hand, corticosteroids, particularly glucocorticoids, act by pro-inflammatory cytokine gene expression, suppressing the immune function and reducing the synthesis of various inflammatory mediators. These agents are effective in managing both acute and chronic inflammatory conditions [5,6]. Glucocorticoids include systemic agents such as prednisone, prednisolone, methylprednisolone, dexamethasone, betamethasone, triamcinolone, deflazacort, budesonide, and beclomethasone, as well as topical agents such as clobetasol, fluocinolone acetonide, and hydrocortisone.

However, the prolonged use of NSAIDs is associated with a significantly increased risk of serious adverse effects across multiple organ systems. The most frequent and clinically relevant complications include gastrointestinal injury, elevated cardiovascular risk, renal impairment, and hepatotoxicity [7,8,9,10]. Similarly, despite their effectiveness, the long-term use of glucocorticoids is also linked to a wide range of adverse effects, including immunosuppression, metabolic and endocrine disturbances, musculoskeletal complications, neuropsychiatric symptoms, and gastrointestinal disorders [11,12].

Clinical data further underline these concerns. Epidemiological studies estimate that up to 2–4% of chronic NSAID users may develop complicated gastric ulcers or gastrointestinal bleeding, with older adults being at higher risk [8]. Diclofenac has also been associated with acute hepatitis and marked elevations in transaminase levels in up to 15% of patients [10]. In the case of glucocorticoids, prolonged use may lead to secondary adrenal insufficiency as well as a significant increase in the risk of vertebral and hip fractures [11].

Due to these limitations, the long-term use of conventional anti-inflammatory drugs is often restricted, prompting the search for alternative therapeutic strategies. One promising approach involves the use of natural bioactive compounds with anti-inflammatory properties.

### 1.2. Bioactive Compounds: Emerging Anti-Inflammatory Agents

The search for safer therapeutic alternatives has driven increasing interest in natural bioactive compounds. Bioactive compounds (BCs) are naturally occurring molecules found in plants, which contain about 80% of all secondary metabolites. However, these compounds also occur in animals, fungi, and microorganisms, and they exert beneficial effects on health beyond basic nutrition. BCs are characterized by their ability to modulate key biological and physiological processes thanks to their chemical structure and biofunctional properties. Among their most notable activities are antioxidant, antimicrobial, antimetabolic, and immunomodulatory activities, with their anti-inflammatory potential being particularly well recognized [13].

BCs are generally classified according to their chemical structure and biological origin. Major classes include phenolic compounds (e.g., flavonoids, phenolic acids, and tannins), terpenes and terpenoids, alkaloids, glycosides, bioactive lipids, sulphur-containing compounds, and bioactive peptides. These compounds may be lipophilic or hydrophilic, with varying distribution in nature, food content, and biological activity in the human body [14].

Among these classes, phenolic compounds are especially abundant in plant-derived foods. They are characterized by the presence of one or more hydroxyl groups (-OH) attached to an aromatic benzene ring. One of the most studied subclasses of phenolics is flavonoids, which share a common structural scaffold known as the flavan nucleus, composed of 15 carbon atoms arranged in three rings (C6–C3–C6), designated as rings A, B, and C. Depending on the oxidation state and structural features of the central C ring, flavonoids are divided into six main subgroups: flavones, flavonols, flavanols, flavanones, isoflavones, and anthocyanins. Structural diversity within these subgroups arises mainly from variations in hydroxylation, methoxylation, prenylation, and glycosylation patterns [15]. Phenolic acids are generally grouped into two categories based on their carbon skeleton: benzoic acid derivatives and cinnamic acid derivatives. Another key class of dietary polyphenols is tannins, which are typically subdivided into hydrolyzable and condensed tannins. Hydrolyzable tannins consist of a central polyol esterified with gallic acid or hexahydroxydiphenic acid. Their substantial structural variability stems from the many possible oxidative linkages between constituent units [16].

Terpenes and terpenoids are composed of repeating isoprene units (C_5_H_8_). Depending on the number of these units, they are classified as monoterpenes (C10), sesquiterpenes (C15), diterpenes (C20), among others. Terpenes are pure hydrocarbons, while terpenoids contain additional functional groups such as alcohols, ketones, or epoxides that enhance their biological activities [17]. These compounds are generally lipophilic and can modulate inflammatory responses by acting on membrane-bound receptors or through intracellular lipid-mediated mechanisms [18]. Alkaloids, by contrast, are characterized nitrogen-containing heterocyclic rings, typically derived from amino acids [19]. This chemical architecture allows them to bind neuronal or enzymatic receptors with high affinity, thereby exerting immunomodulatory and anti-inflammatory effects [20,21]. Other relevant classes include glycosides, bioactive lipids, sulphur-containing compounds, and bioactive peptides. Glycosides consist of an aglycone moiety linked to one or more sugar units via glycosidic bonds, which enhances their solubility and bioavailability [22].

Bioactive lipids, such as polyunsaturated fatty acids (PUFAs), are composed of long hydrocarbon chains with multiple double bonds. These structures are precursors to anti-inflammatory lipid mediators [23]. Sulphur-containing compounds, such as glucosinolates, possess structures rich in thiol and sulfonate groups that are converted into bioactive isothiocyanates upon hydrolysis [24]. Finally, bioactive peptides are short oligopeptides derived from protein hydrolysis, whose specific amino acid sequences enable them to inhibit inflammatory enzymes or modulate immune responses [25].

**Figure 1:**
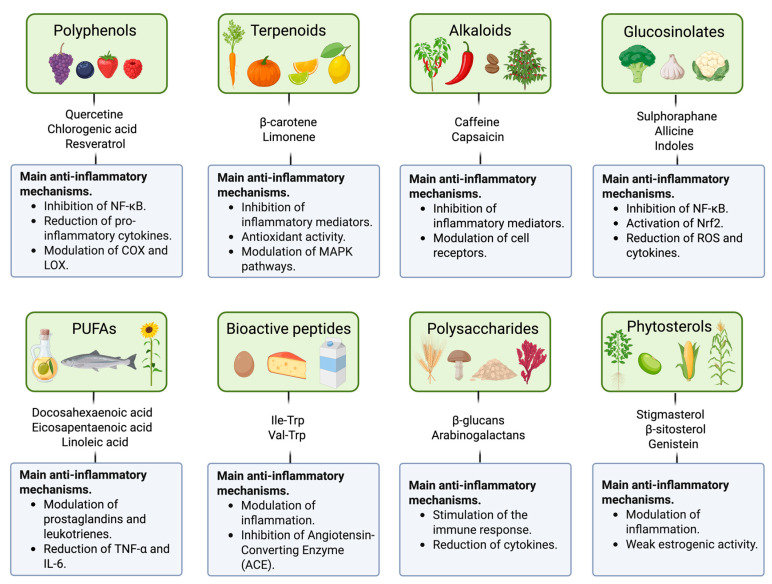
Main sources and classification of BCs with anti-inflammatory properties. Figure created from information obtained in Hang et al. [13] and Samiappan et al. [26] using BioRender.com.

Collectively, BCs exert their anti-inflammatory effects through multiple mechanisms. These include inhibition of pro-inflammatory cytokine expression (e.g., TNF-α, IL-6, and IL-1β), reduction in oxidative stress, suppression of key signalling pathways such as NF-κB or MAPK, and modulation of enzymes like COX and nitric oxide synthase (NOS) (Figure 1). Their multi-targeted mode of action provides them with high therapeutic potential, especially as an alternative or complement to conventional anti-inflammatory drugs, whose adverse effects limit long-term use [13,26].

### 1.3. Extraction Methods for Bioactive Compounds

**Figure 2:**
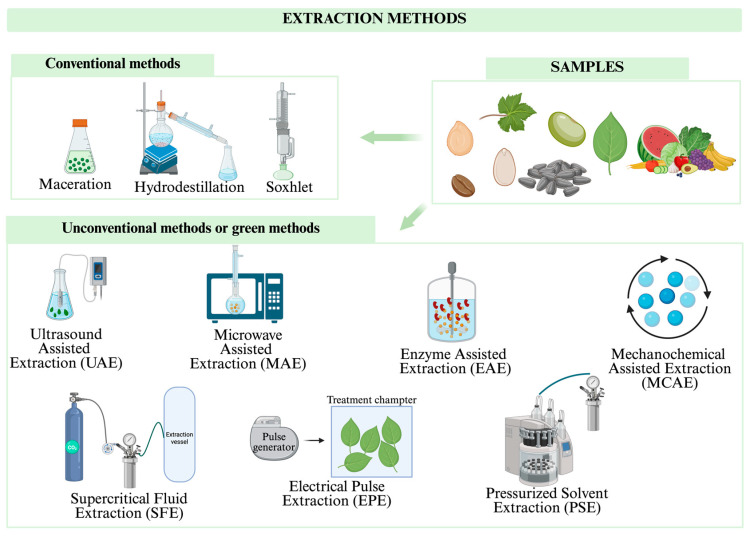
Extraction methods for bioactive compounds. Created with https://BioRender.com.

BCs are primarily obtained from natural sources such as plants, fruits, seeds, algae, and agricultural by-products. A range of extraction techniques is employed to preserve their structural integrity, biological activity, and purity. These methods fall broadly into two categories (Figure 2): (a) conventional extraction methods and (b) unconventional, innovative, or “green” extraction methods, which are designed to improve efficiency, reduce solvent use, and minimize environmental impact.

Conventional extraction methods include (a) maceration, which involves leaving the material in contact with a solvent, such as water or ethanol, for an extended period of time, allowing the compounds to dissolve into the solvent [27,28]; (b) Soxhlet extraction, which uses a heated solvent that repeatedly circulates through the plant material, making it especially effective for compounds with low water solubility [28]; (c) hydrodistillation, which is mainly used for essential oils and involves the application of water vapour to the plant material [29,30].

In the case of unconventional, innovative, or “green” methods, the following can be found: (a) Ultrasound Assisted Extraction (UAE), which employs ultrasonic waves to break down cell walls and release BCs more quickly and efficiently, with reduced solvent consumption [31,32,33]; (b) Microwave-Assisted Extraction (MAE), which heats the solvent and material using microwave energy, accelerating the extraction process [33,34]; (c) Supercritical Fluid Extraction (SFE), which uses supercritical carbon dioxide to extract compounds at moderate temperatures without leaving toxic residues. This method is especially useful for heat-sensitive compounds [35]; (d) electrical pulse extraction involves applying high-voltage electrical fields to increase the permeability of cell membranes and facilitate the outflow of BCs [36,37,38]; (e) enzyme-assisted extraction uses specific enzymes to degrade cell walls and release compounds of interest, improving yield and selectivity [39,40,41]; (f) pressurized solvent extraction improves solubility and selectivity, allowing purer, more concentrated extracts to be obtained [42,43]; (g) a new technique called Mechanochemical-Assisted Extraction (MCAE) has emerged in recent years. It is presented as an ecological and efficient strategy for extracting bioactive compounds, fibres, and other valuable molecules from a wide range of biomass sources [44,45,46].

In fact, articles using this technique alongside Natural Deep Eutectic Solvents (NADES) have already been published. One example is the article by Cubero-Cardoso et al., in which different systems are used to extract bioactive products from olive leaves [47].

Following extraction, BCs typically undergo purification processes, such as chromatography or resin adsorption, to obtain more concentrated and biologically active fractions suitable for downstream applications.

### 1.4. Deep Eutectic Solvents as Extraction Media

Green extraction technologies are a set of methods and strategies developed according to the principles of green chemistry. Their goal is to efficiently, safely, and sustainably obtain target compounds from natural sources. These techniques aim to minimize environmental impact by reducing energy consumption, employing renewable or low-toxicity solvents, and designing processes that limit waste generation [30]. In this context, Deep Eutectic Solvents (DESs) have emerged as an innovative approach to green extraction. They offer advantages over conventional organic solvents due to their low toxicity, biodegradability, and the ability to adjust their physicochemical properties for the optimized extraction of specific BCs. Thus, the use of DESs is fully aligned with the objectives and conceptual framework of green extraction, promoting the transition toward more environmentally responsible scientific and industrial practices [48,49]. Their effectiveness stems from their excellent solubilizing capacity and tunable characteristics. Abranches et al. defined DESs as a subclass of eutectic solvents (mixtures that are in the liquid phase at a specified temperature, even though at least one of their components would typically be a solid under those conditions) characterized by enthalpically driven negative deviations from ideal thermodynamic behaviour [50]. These solvents consist of a combination of two or more compounds that can stablish molecular interactions between the components, such as hydrogen bonding, van der Waals forces, or electrostatic interactions, which disrupt the crystalline structure and stabilize the liquid phase [51]. Depending on the nature of their component (whether hydrophobic or hydrophilic) DESs can be classified as type I (metal halide and a quaternary ammonium salt), type II (hydrated metal halide and a quaternary ammonium salt), type III (quaternary ammonium salt and a Hydrogen Bond Donor (HBD), type IV (metal halide and HBD), and type V (a neutral Hydrogen Bond Acceptor (HBA) and a neutral HBD) [52,53]. There are other classifications or sub(groups) of DESs attending to (sub)groups according to the origin/source of their components or their purpose, i.e., Natural Deep Eutectic Solvents (NADESs) or THEDESs (Therapeutic Deep Eutectic Solvents). The DES mixtures have demonstrated good performance in extraction processes thanks to their high solvent capacity [54,55,56]. DESs are relevant for extracting purposes since their physicochemical properties can be tailored by adjusting the HBDs and HBAs, allowing for optimized extraction performance depending on the target compound.

Several factors must be considered when selecting an appropriate DES system. Firstly, the combination of HBA and HBD is crucial, such as choline chloride (ChCl) combined with organic acids or sugars. Depending on the combinations selected, either hydrophobic or hydrophilic mixtures will be obtained. Secondly, the compounds to be extracted and their characteristics must be determined. Polar compounds such as polyphenols, flavonoids, and alkaloids, for example, prefer hydrophilic DESs due to their high polarity and ability to form hydrogen bonds. This improves solubility and therefore their extraction capacity. For acidic compounds and antioxidants, combinations with organic acids favour interaction with carboxyl and hydroxyl functional groups, thereby improving the capture of these BCs. Low-molecular-weight compounds and hydroxyl/carbonyl functional groups prefer systems formed by ChCl and sugars as these allow extractions that are compatible with pharmaceutical and food applications due to their low toxicity and biodegradability. In the case of polar or aromatic compounds, hydrophobic DESs, such as those based on phenols, are preferred as they allow the extraction of less polar compounds, although care must be taken regarding toxicity and the final application. Finally, consider the molecular weight, polarity, and functional groups. To maximize solubility, the polarity of the systems should be close to that of the target compounds, i.e., polar DESs for polar compounds and hydrophobic DESs for non-polar compounds. The solute’s functional groups must complement the DES’s hydrogen bonding capacity to improve their interaction and, therefore, extraction [57,58].

## 2. Applications of Deep Eutectic Solvents in the Extraction of Anti-Inflammatory Bioactive Compounds

This review is distinguished by its clear distinction between ChCl-based systems and other types of DESs that do not contain ChCl. Unlike other reviews that address a wide range of BCs, our work focuses exclusively on those with anti-inflammatory activity. This allows us to delve deeper into the relationship between solvent selection, extraction techniques, and the efficiency with which anti-inflammatory molecules can be obtained, providing a more specific and applied view of the topic.

This section presents findings from the literature review regarding the extraction of BCs with DESs as extraction media. The results are discussed according to the eutectic system and its components used to extract the bioactive compounds. Firstly, we will study polar DESs and, secondly, apolar DESs. Within the polar DES category, one component stands out: choline chloride (ChCl). This quaternary ammonium salt plays a central role in DES formulation due to its high polarity, low toxicity, biodegradability, and strong capacity to form hydrogen bonds with a wide range of HBDs. A summary of the studies reviewed about the extraction of BCs using ChCl-based DESs is provided in Table 1.

Certainly, ChCl is one of the most used HBA in the formulation of DESs employed as extraction media for compounds with potential anti-inflammatory activity [59,60,61,62,63,64], commonly found in binary DESs, where it is combined with sugar [65], urea, polyalcohols such as glycerol [65] or 1,2-propanediol [66], or organic acids [65,67]. Additionally, ChCl is also present in ternary DESs used for extraction purposes [59]. In these systems, the third component is most often water [59,61,68], although other ternary DESs have been described, for example, mixtures of ChCl, citric acid, and glycerol [62] for extracting potential anti-inflammatory bioactivity substances from natural sources.

**Table 1:** Extraction of bioactive compounds using ChCl-based DESs: compound families, species, DES types, extraction methods, and yields.

Bioactive Molecule	Type of Bioactive Molecule	Specie	DES		Extraction Method	Extraction (Yield (mg/g))	Reference
			Components	Ratio			
Curcumin	Phenol (curcuminoids)	*Curcuma longa*	ChCl–Glycerol–Water	1–1–5	Stirring-assisted extraction	2.1	[59]
			ChCl–Fructose–Water	1–1–5		0.7	
			ChCl–Glycerol–Water	1–2–5		0.5	
			ChCl–Glucose–Water	1–1–3		1.6	
			ChCl–Glycerol–Water	1–1–2		2.0	
			ChCl–Citric acid–Water	1–2–5		0.4	
			ChCl–Citric acid–Water	2–1–5		0.5	
			ChCl–Glycerol	1–1		0.4	
			ChCl–Glycerol	1–2		0.4	
			ChCl–Glycerol	1–3		0.3	
			Glycerol–Urea–Water	1–1–2		0.1	
			ChCl–Xylitol–Water	1–1–5		0.5	
			ChCl–Citric acid–Water	1–2–3		0.4	
			ChCl–Lactic acid–Water	1–2–5		1.4	
			ChCl–Citric acid–Water	1–1–5		2.0	
			ChCl–Urea–Water	1–2–5		0.7	
			ChCl–Glycerol–Citric acid–Water	0.5–2–0.5–5		2.7	
			ChCl–1,2-propanediol–Water	1:1:1		3.1	
Ascorbic acid, citric acid, cinnamic acid, gallic acid, catechin, quercetin, coumaric acid, tartaric acid, catechol, curcumin, pyrogallic acid, caffeic acid, tannic acid, etc.	Phenolic compounds	*Clematis fammula* L. Leaves and *Pistacia lentiscus* L. Fruits	ChCl–Acetic acid	1:2	UAE	Not given in detail (>80%)	[60]
Anthocyanin-rich fraction; non-anthocyanin phenolic fraction. 42% anthocyanins: delphinidin, cyanidin, petunidin, malvidin, etc.	Polyphenols (anthocyanins)	*Vaccinium corymbosum* L. *x Vaccinium* *darrowii* Camp.	ChCl–Glycerol–Citric acid	0.5:2:0.5	UAE	11.4 mg/g (4.57 mg of total polyphenols/mL, with the extract coming from a solution containing ~0.4 g/mL of freeze-dried powder)	[62]
Protocatechuic acid, chlorogenic acid isomer, chlorogenic acid, caffeine acid, p-coumaric acid, rutin	Polyphenols	Dried seeds (fruits) of *Coriandrum sativum* L.	ChCl–Glucose	1–1	UAE	Yield data obtained (mg/g) for each compound using ChCl–Urea and ChCl–glucose, respectively– - Chlorogenic acid isomer– 0.537 mg/g and 0.184 mg/g. - Chlorogenic acid– 0.453 mg/g and 0.152 mg/g. - Caffeic acid– 0.093 mg/g and 0.269 mg/g. - p-coumaric acid– 0.030 mg/g and 0.057 mg/g. - Rutin– 0.766 mg/g and 0.235 mg/g	[63]
			ChCl–Glycerol	1–1			
			ChCl–Urea	1:1			
			ChChl–Citric acid	1:1			
naringenin, naringin, hesperidin, vitexin, apigenin, luteolin, and luteolin 7-glucoside, quercetin, quercitrin, rutin, kaempferol, phloretin, phloridzin, genistein	Flavonoids	*Lippia graveolens*	ChCl–Ethyleneglycol–Water	1–4–30% Water *v*/*v*	Maceration, UAE, Supercritical Fluid	39.5	[64]
			ChCl–Glycerol–Water	1–4–30% Water *v*/*v*		39.4	
			ChCl–Lactic acid–Water	1–4–30% Water *v*/*v*		37.3	
	Phenolic compounds		ChCl–Ethyleneglycol–Water	1–4–30% Water *v*/*v*		100.1	
			ChCl–Glycerol–Water	1–4–30% Water *v*/*v*		123.6	
			ChCl–Lactic acid–Water	1–4–30% Water *v*/*v*		126.1	
Bilberry	Anthocyanins	*Vaccinium myrtillus*	ChCl–Lactic acid	1–2	UAE	2.48	[65]
			ChCl–Citric acid–Water	1–1–2		1.78	
			ChCl–Malic acid–Water	1–1–2		2.24	
			ChCl–Tartaric acid	1–2		1.25	
			ChCl–Glycerol	1–2		1.90	
			ChCl–1,2-propanediol	1–3		2.00	
			ChCl–Sorbitol	1–1		2.03	
			ChCl–Glucose–Water	2–1–1		1.74	
			ChCl–Fructose–Water	2–1–1		1.64	
			ChCl–Urea	1–2		0.92	
Comfrey	Rosmarinic acid	*Symphytum officinale*	ChCl–Glycerol	1–2	UAE	<0.5	[69]
			ChCl–Urea	1–2			
			ChCl–Sucrose	1–2			
Turmeric—curcumin	Curcuminoids	*Curcuma longa*	ChCl–L	1–1	DoE-Optimized (Box–Behnken Designed) Supercritical CO_2_ Extraction.	13.8	[70]
			ChCl–Citric acid	1–1		8.22	
			ChCl–Urea	1–2		12.5	
			ChCl–Propylene glycol	1–2		23.1	
Hydroxytyrosol, tyrosol, Hy-Ac, oleacein (Hy-EDA), oleocanthal, 1-acetoxypinoresinol, Hy-EA, luteolin, ty-EA, apigenin	Phenolic compounds	*Olea europaea* (Olive oil)	ChCl–Glycerol	1–2	Shaking extraction at 40 °C	0.150	[71]
			ChCl–Lactic acid	1–3		0.073	
			ChCl–Urea	1–4		-	
			ChCl–Sucrose	1–1		0.140	
			ChCl–Sucrose	4–1		0.058	
			ChCl–1,4-butanediol	1–5		0.120	
			ChCl–Xylitol	2–1		0.190	
			ChCl–1,2-propanediol	1–1		0.170	
			ChCl–Malonic acid	1–1		0.085	
			ChCl–Urea–Glycerol	1–1–1		-	
a-Mangostin	Polyphenol	Mangosteen (*Garcinia mangostana* L.)	ChCl–1,2-propanediol	1–3	Shaking-assisted extraction at room temp.	5.20	[72]
Rutin	Flavonoid	*Sophora japonica*	ChCl–1,4-butanediol–Water	1–4–20% Water *v*/*v*	Stirring-assisted solid–liquid extraction	170	[68]
			ChCl–Acetamide–Water	1–2–20% Water *v*/*v*		170	
			ChCl–Citric acid–Water	1–1–20% Water *v*/*v*		100	
			ChCl–Sorbitol–Water	1–1–20% Water *v*/*v*		100	
			ChCl–Ethyleneglycol–Water	1–2–20% Water *v*/*v*		180	
			ChCl–Fructose–Water	5–2–20% Water *v*/*v*		60	
			ChCl–Glucose–Water	5–2–20% Water *v*/*v*		100	
			ChCl–Glycerol–Water	1–2–20% Water *v*/*v*		150	
			ChCl–Levunillic acid–Water	1–2–20% Water *v*/*v*		200	
			ChCl–Malic acid–Water	1–1–20% Water *v*/*v*		100	
			ChCl–Malonic acid–Water	1–1–20% Water *v*/*v*		200	
			ChCl–Maltose–Water	5–2–20% Water *v*/*v*		50	
			ChCl–Oxalic acid–Water	1–1–20% Water *v*/*v*		100	
			ChCl–p-toluenesulfonic acid–Water	1–1–20% Water *v*/*v*		100	
			ChCl–Sucrose–Water	5–2–20% Water *v*/*v*		50	
			ChCl–Tartaric acid–Water	2–1–20% Water *v*/*v*		120	
			ChCl–Triethylene glycol–Water	1–4–20% Water *v*/*v*		194	
			ChCl–Urea–Water	1–2–20% Water *v*/*v*		180	
			ChCl–Xylitol–Water	1–1–20% Water *v*/*v*		170	
			ChCl–Xylose–Water	1–1–20% Water *v*/*v*		50.0	
Tocopherols and tocotrienols	Tocols	*Elaeis guineensis* Crude Palm Oil	ChCl–Malonic acid	1–3	Liquid–liquid extraction	0.018	[67]
Apigenin	Flavonoids	*Cajanus cajan* (Pigeon pea) roots	1,6-hexanediol–ChCl–Water	7–1–30% Water *v*/*v*	MAE	0.220	[73]
Genistin	Polyphenols		1,6-hexanediol–ChCl–Water	7–1–30% Water *v*/*v*		0.450	
Genistein	Polyphenols		1,6-hexanediol–ChCl–Water	7–1–30% Water *v*/*v*		0.620	
Chimaphilin	Phenols	*Pyrola incarnata* Fisch	ChCl–1,4-butanediol–Water	1–4–30% Water *v*/*v*	MAE	0.350	[74]
Hyperin						1.63	
20-O-galloylhyperin						4.96	
Quercetin						0.041	
Quercetin-Orhamnoside						0.089	
Chimaphilin						0.350	
Rosmarinic acid	Polyphenol	*Prunella vulgaris*	ChCl–Ethylene glycol–Water	1–4–36% Water *v*/*v*	Ultrasonic	3.66	[75]
Salviaflaside			ChCl–Ethylene glycol–Water	1–4–36% Water *v*/*v*	Ultrasonic	1.05	[75]
Rutin	Flavonoid	*Fagopyrum esculentum* (Buckwheat)	ChCl–Glycerol–Water	1–1–20% Water *v*/*v*	UAE	9.50	[76]
Rosmarinic acid	Phenolic compounds	*Salvia rosmarinus*	ChCl–Urea	1–1	Liquid–liquid extraction and ultrasonic water bath	15.7	[77]
		*Satureja hortensis*	ChCl–Citric acid	1–1		11.5	
		*Lavandula angustifolia*	ChCl–1,2-propanediol	1–1		1.99	
		*Salvia officianalis*	ChCl–1,2-propanediol	1–1		6.57	
		*Melissa officinalis*	ChCl–Urea	1–1		19.5	
		*Origanum vulgare var.h*	ChCl–1,2-propanediol	1–1		4.69	
		*Ocimum basilicum*	ChCl–Urea	1–1		4.50	
		*Thymus serpyllum*	ChCl–1,2-propanediol	1–1		12.4	
Rosavin	Flavonoids, polyphenols	*Rhodiola rosea* L.	ChCl–Malonic acid	1–1	UAE	NADESs with malonic and tartaric acids showed the highest efficiency in extracting polyphenols, outperformed reference solvents by 10–20 mg GAE/g and reached a value of 160 mg GAE/g.	[78]
			ChCl–Malic acid	1–1			
			ChCl–Citric acid	1–1			
			ChCl–Tartaric acid	2–1			
Orientin and vitexin	Flavonoids	*Trollius ledebouri*, (dry flowers)	ChCl–1,3 butanediol	1–2	UAE	Orientin 13.5 mg/g, vitexin 1.30 mg/g	[79]
			ChCl–1,4 butanediol	1–3		Orientin 14.5 mg/g, vitexin 1.25	
			ChCl–Ethylenglycol	1–2		Orientin 13.0 mg/g, vitexin 1.20 mg/g	
			ChCl–Glycerine	1–2		Orientin 12.5 mg/g, vitexin 1.00 mg/g	
			ChCl–Oxalic acid–Ethylenglicol	1–1–2		Orientin 16.5 mg/g, vitexin 1.50 mg/g	
			ChCl–Sorbitol	1–1		Orientin 12.5 mg/g, vitexin 1.40 mg/g	
			ChCl–Malonic acid	1–1		Orientin 14.2 mg/g, vitexin 1.30 mg/g	
			ChCl–Lactic acid	1–1		Orientin 14.5 mg/g, vitexin 1.40 mg/g	
			ChCL–Citric acid	1–1		Orientin 16.0 mg/g, vitexin 1.6 0 mg/g	
			ChCl–Malic acid	1–1		Orientin 14.5 mg/g, vitexin 1.25 mg/g	
			ChCl–Fructose–water	2–1–1		Orientin 14.0 mg/g, vitexin 1.60 mg/g	
			ChCl–Glucose	2–1		Orientin 15.0 mg/g, vitexin 1.40 mg/g	
			ChCl–Urea	1–2		Orientin 12.5 mg/g, vitexin 1.40 mg/g	
Phenolic compounds	Polyphenols	Olive leaves (OL) of the Picual variety	ChCl–oxalic acid	1–1	MCAE	1.7	[47]
			ChCl–oxalic acid	2–1		0.6	
			ChCl–urea	1–2		0.8	
			ChCl–fructose–water	5–2–5		0.6	
			ChCl–lactic acid	1–2		0.7	
			ChCl–glucose	3:1		1.3	

The most commonly employed methods for extracting BCs with anti-inflammatory potential using ChCl-based DESs include conventional liquid–liquid extraction via stirring or shaking [59,60], as well as assisted techniques such as ultrasound-assisted extraction [60,62] and microwave-assisted extraction [61]. For each of these approaches, various experimental parameters are investigated, including extraction temperature, exposure time, and other factors [67,80]. In some cases, ultrasound-assisted solid–liquid extraction has also been reported. For example, Jovanović et al. used bilberry fruits (*Vaccinium myrtillus* L.) to extract anthocyanins with ChCl-based DESs, concluding that the best results were obtained using a ChCl–sorbitol (1:1) system [65].

Regarding the BCs with anti-inflammatory properties extracted using ChCl-based DESs, it is noteworthy that most are phenolic compounds or polyphenols [75,80]. Another major class includes flavonoids [68]. Tocols [67] and anthocyanins [65] have also been efficiently extracted with ChCl-based DESs. In the study by Tsvetov et al., the total polyphenols content (expressed in mg of gallic acid equivalent) extracted from *Rhodiola rosea* L. using ChCl-based DESs combined with organic acids, such as malonic, malic, citric, and tartaric acids, reached high levels (up to 248 mg/g with malonic acid DESs) [78]. Focusing on specific compounds, curcumin and its derivatives have been extensively studied due to their anti-inflammatory properties [59,70]. Comparative studies show that binary DESs composed of ChCl and lactic acid (1:1), citric acid (1:1), urea (1:2), or propylene glycol (1:2) achieved higher curcumin extraction yields from *Curcuma longa* than ternary ChCl-based DESs containing water. While ternary DESs yielded a maximum of 3.2 mg/g [60], binary DESs produced yields ranging from 8 to 23 mg/g [70], with the ChCl–propylene glycol (1:2) system exhibiting the highest performance [70]. Another study employed a DES composed of ChCl and acetic acid (1:2) to satisfactorily extract anti-inflammatory compounds, including curcumin, from *Clematis flammula* L. leaves and *Pistacia lentiscus* L. fruits, though quantitative comparisons were not possible due to the lack of specific data [60]. The polyphenol rosmarinic acid has been extracted using ChCl-based DESs combined with glycerol, urea, and sucrose, all at a 1:2 molar ratio [69]. In all these cases, the extraction yields were below 0.5 mg/g of dry residue. These values are lower than those obtained when betaine-based DESs were used under the same extraction conditions [69]. Other studies have reported higher rosmarinic acid yields, such as from *Prunella vulgaris* using ChCl–ethylene glycol (1:4) with 36% *v*/*v* water, reaching up to 3.6 mg/g [75]. In Juric et al., ChCl-based DESs (1:1 molar ratio) with urea, citric acid, or 1,2-propanediol were used to extract rosamarinic acid form *Salvia rosmarinus*, *Satureja hortensis*, *Lavandula angustifolia*, *Salvia officianalis*, *Melissa officinalis*, *Origanum vulgare var.hirtum*, *Ocimum basilicum*, and *Thymus serpyllum* achieving yields from 4.50 to 19.52 mg/g [77]. These findings suggest that both the choice of HBD and HBA significantly influence rosmarinic acid extraction efficiency [69].

When combined with 1,2-propanediol in a 1:3 molar ratio, ChCl is capable of extracting more than 5.2 mg/g of α-mangostin from mangosteen (*Garcinia mangostana* L.) [72]. In other examples, low extraction yields, below 1 mg/g, have been reported when using ChCl + urea or glucose DESs for the recovery of protocatechuic acid, chlorogenic acid, caffeic acid, and *p*-coumaric acid from dried seeds (fruits) of *Coriandrum sativum* L. [63]. The extraction of phenolic anthocyanins from *Vaccinium myrtillus* has also been studied using ternary ChCl-based DESs that included water. In none of the cases examined did the results surpass those obtained using methanol extraction [65]. Similarly, low yields (below 0.2 mg/g) were obtained in the extraction of several phenolic compounds (tyrosol or hydroxytyrosol and related compounds, oleacein, oleocanthal, 1-acetoxypinoresinol, luteolin, and apigerin) from olive oil using common binary ChCl-based DESs with glycerol, urea, xylitol, sucrose, 1,4-butanediol, 1,2-propanediol, and malonic or lactic acid [71]. From crude palm oil, tocols were also extracted with very low efficiency (less than 0.02 mg/g) using 1:3 ChCl–malonic acid DESs [67].

Diols are common HBDs used in combination with ChCl in DESs for extraction purposes. For example, DESs composed of ChCl and 1,6-hexanediol in a 7:1 molar ratio, with 30% water (*v*/*v*), have been used for microwave-assisted extraction of flavonoid-type polyphenols from pigeon pea (*Cajanus cajan*) roots, yielding 0.221 mg/g of apigenin, 0.449 mg/g of genistein, and 0.617 mg/g of genistin, respectively [73]. Similarly, in another study, DESs formed by ChCl and 1,4-butanediol (1:4) and water (30%) was used to extract polyphenolic flavonoids from *Pyrola incarnata Fisch* using MAE method. However, the extraction performance was low (less than 0.1 mg/g) for quercetin and quercetin–orhamnoside, but higher for 20-O-galloylhyperin, reaching nearly 5 mg/g [74]. The polyphenol salviaflaside was also extracted from *Prunella vulgaris* using a DES composed of ChCl–ethylene glycol (1:4) with 36% water; however, the extraction yield was relatively low, at approximately 1 mg/g of dry plant material [75].

Paying closer attention to flavonoids, several studies have investigated the extraction of rutin from various plant matrices such as buckwheat [68,76] and *Sophora japonica* using aqueous ternary ChCl-based DESs (with 20% water in all cases). The results indicate high extraction yields from *Sophora japonica*, reaching up to 200 mg/g when using DESs containing levulinic acid (1:2) or malonic acid (1:1) as HBDs [68]. In contrast, the yield from buckwheat was significantly lower, with values around 10 mg/g using the only DES studied, ChCl–glycerol (1:1) with 20% water [69]. In another study, the total flavonoid content, expressed as quercetin equivalents, extracted from *Lippia graveolens* was higher when ChCl-based DESs were used in combination with ethylene glycol, glycerol, or lactic acid (in a 1:4 molar ratio with 30% water), compared to conventional solvents [64]. Specifically, the yields were approximately twice as high as those obtained with water, 1.5 times higher than with methanol, and seven times higher than with supercritical CO_2_. In the same study, the extraction yield of total phenolic compounds (TPCs) using DESs also outperformed the conventional solvents, with yields 4.5 times higher than with water, twice as high as with methanol, and again, seven times greater than with supercritical CO_2_ [64]. Finally, it is worth mentioning a study that investigated the ultrasonic-assisted extraction of flavonoids from dried flowers of *Trollius ledebouri* using ChCl- and betaine-based DESs with various HBD compositions. The results indicated that both types of DESs exhibited similar extraction performances, yielding between 12 and 16 mg/g for orientin and between 1.16 and 1.6 mg/g for vitexin [79].

Finally, the article by J. Cubero-Cardoso et al. presented five systems consisting of ChCl, oxalic acid, urea, fructose, and water; lactic acid and glucose; and different molar ratios of these substances. BCs from olive leaves were obtained using the MCAE method, after which the total amount of sugars, uronic acid, TPCs, antioxidant capacity, and different phenolic compounds were analyzed. It was observed that the ChCl–oxalic acid system in a 1:1 ratio produced the highest yield of phenolic compounds (1.7 mg/g), followed by ChCl–glucose 3:1, ChCl–urea 1:2, ChCl–lactic acid 1:2, ChCl–oxalic acid 2:1 and ChCl–fructose–water 5:2:5. It should be noted that the yield decreased when the proportions of ChCl and oxalic acid were modified [47].

On the other hand, NADESs are a natural alternative to ChCl designed by combining naturally occurring HBAs and HBDs, such as primary metabolites (e.g., amino acids, sugars, and organic acids), betaine, L-proline, and glycerol. These mixtures enable the polarity, viscosity, and extractive capacity of the system to be modulated according to the target compound. Table 2 lists results obtained from the review of studies involving extraction of BCs using DESs consisting of HBAs other than ChCl.

In the article published by Hee Nam et al., the NADES system consisting of betaine (HBA) and 1,2-propanediol (HBD) in a 1:2 molar ratio was used to extract BCs with anti-inflammatory properties, such as geraniin, corilagin, ellagic acid, and gallic acid, from the peel of rhambutan (*Nephelium lappaceum* L.). The extraction was performed via liquid–liquid extraction, yielding 54.29 mg/g [81].

Phenolic acids, flavonoids, and tannins have also been extracted from blackcurrant (*Ribes nigrum* L.) leaves using NADESs. In this study, Solcan et al. prepared 39 systems consisting of either L-proline or ChCl as the HBA, and either polyethylene glycol, lactic acid, or glucose as the HBD. The TPC, total amount of flavonoids (TFC), and total antioxidant activity were determined. Ultrasound-assisted extraction and ultra-turrax extraction (UTE) were used for different durations. The highest TPC yields were obtained with L-proline–lactic acid and ChCl–glucose, with values between 400 and 490 mg gallic acid equivalents (GAE)/g. For TFC, the highest values were obtained for L-proline–lactic acid and L-proline–glucose, with values of approximately 14 mg quercetin equivalents per gram of dry weigh (QE/g dw) [82].

Wils et al. conducted a study in which 20 polar and apolar NADES systems for the extraction of pigments and free fatty acids from spirulina (*Arthrospira platensis*) were prepared and screened. These systems consisted of betaine, glucose, lactic acid, menthol, and derivatives of octanoic and nonanoic acids at different molar ratios. The NADESs that allowed the most metabolites to be extracted consisted of glycerol and glucose. Furthermore, the system consisting of fatty acids showed the greatest selectivity towards free fatty acids [83].

Kaoui et al. prepared three systems consisting of lactic acid with glucose (5:1), sodium acetate (3:1), and glycine (3:1) to extract polyphenolic compounds found in *Mentha pulegium* L. For the extraction, ultrasound-assisted extraction was combined with NADESs. They determined that all NADESs extracted more efficiently than methanol, obtaining the highest yields for the lactic acid–sodium acetate (3:1) system with values of 173.35 ± 0.02 mg gallic acid equivalents (GAE)/g dry weight (d.w.) of polyphenols [84].

Another important study was conducted on the extraction of flavonoids from *Amomum villosum* Lour (*A. villosum*) where 20 NADESs (non-aqueous dual-phase extraction systems) derived from betaine and L-proline as the HBA (hydrophilic base atom), using the following HBDs (hydrophilic base donors) prepared for ultrasound-assisted extraction: propylene glycol, glycerol, urea, lactic acid, malic acid, citric acid, glucose, fructose, sorbitol, and xylitol. A greater diversity of flavonoids was observed in the extracts, obtaining a maximum total flavonoid content of 82.22 ± 0.39 mg of rutin equivalent per gram of dry weight (mg Rut/g DW) [85].

Flavonoids have also been extracted from *Flos sophorae* using deep eutectic systems. Different combinations were tested, consisting of L-proline and glycerol (10% in water, *v*/*v*) at molar ratios of 1:2.5, 1:3, 1:3.5, 1:4, and 1:4.5, as well as L-proline and xylitol at ratios of 1:1, 1:1.5, 1:2, 1:3, and 1:4. The best system was found to be that formed by L-proline and glycerol at a molar ratio of 2:5, as this enable the extraction of quercetin and isorhamnetin glycosides by ultrasound-assisted extraction with yields of 100 mg/g and 12 mg/g, respectively [86].

Paradiso et al. prepared a DES consisting of lactic acid, glucose, and water in a 6:1:6 molar ratio, with the aim of extracting phenolic acids from virgin olive oil. Extraction was achieved through a combination of shaking, centrifugation, and filtration. While the extraction yield of each compound is not specified, a regression equation is presented that relates the total phenolic content (mg gallic acid/kg oil) to the wavelengths. This makes it easy to make an approximate determination of the content [87].

Cao et al. published an article demonstrating how DESs can be used to extract BCs from *Artemisia annua* leaves. They tested several systems for extracting artemisinin and found that the system with the highest extraction capacity was methyl trioctyl ammonium chloride–1-butanol (1:4). Ultrasound-assisted extraction was employed, and the yield was higher than that obtained using ether, reaching 7.9936 ± 0.0364 mg/g [88].

Another article studying the extraction of polyphenols from *Carthamus tinctorius* L. (safflower) was published by Dai et al., in which they prepared nine DESs consisting of proline–malic acid–water (1:1:25% water), lactic acid–glucose–water (1:1:25% water) and sucrose–ChCl–water (5:5:25% water). The amount of water was an important factor in the yield of these compounds [80].

## 3. Strengths and Limitations in the Use of DESs for BCs Extraction

This section summarizes the main advantages and limitations of using deep eutectic solvents for extracting anti-inflammatory BCs, as identified in the current literature.

The main advantages of DESs include (a) environmental friendliness and low toxicity [89,90,91]; (b) unique physicochemical properties, such as tunable polarity, which contribute to enhanced extraction yields and selectivity compared to conventional organic solvents; (c) versatility in extracting a broad spectrum of phytochemicals [92]; (d) improved extraction yield and preservation of bioactivity compared to conventional solvents [93]; (e) adaptability, allowing for the selective extraction of specific compounds, which is valuable for pharmaceutical and nutraceutical applications [94]; (f) cost-effective production without requiring significant upfront investment [95]; and (g) identification of novel bioactivities in familiar plant species due to the distinctive chemical composition of DES-based extracts [95].

However, despite being a potential alternative for extracting different biomolecules, DESs also present certain limitations: (a) lack of standardization in the preparation and application of DES [96]; (b) limited data on solvent recyclability and large-scale viability [97]; (c) insufficient toxicological studies to fully assess their safety; (d) available information limited to in vitro studies, with little in vivo or clinical evidence confirming the anti-inflammatory effects of the extracted compounds; (e) limitations for the industrial scale-up due to high viscosity of DESs [81]; (f) more complexity than for traditional solvents in the case of downstream DES-assisted extraction [81]; (g) the economic and regulatory considerations for their industrial implementation also remain unexplored.

## 4. Conclusions

Anti-inflammatory drugs, particularly NSAIDs and corticosteroids, have long been used to control inflammation by targeting specific molecular mechanisms. While effective in the short term, their long-term use is limited due to a wide range of serious adverse effects, including gastrointestinal, cardiovascular, renal, metabolic, and immunosuppressive complications. This limitation has intensified the search for safer therapeutic alternatives. In this context, natural BCs have emerged as promising candidates for managing inflammation more safely. Found in plants, foods, microorganisms, and other natural sources, BCs exhibit significant anti-inflammatory activity through multitarget mechanisms. Structurally diverse, BCs are classified into several major groups, including phenolic compounds (e.g., flavonoids and tannins), terpenes, alkaloids, glycosides, bioactive lipids, sulphur-containing compounds, and peptides.

To harness the benefits of BCs effectively, extraction techniques play a crucial role. While conventional methods such as maceration, Soxhlet extraction, and hydrodistillation remain widely used, they often require high solvent volumes and long processing times. In contrast, innovative “green” extraction technologies, such as UAE, MAE, supercritical fluid, enzyme-assisted, or pulsed electric field extraction, offer enhanced efficiency, selectivity, and sustainability. These methods not only improve extraction yields but also help preserve the structural integrity and biological activity of sensitive compounds.

Ultimately, the combination of natural BCs and environmentally friendly extraction technologies represents a compelling strategy for developing safer, more effective, and sustainable anti-inflammatory agents. In this sense, the use of DESs is gaining attraction in the field thanks to the numerous advantages and extraction potential of these solvents.

The reviewed literature highlights the significant role of ChCl-based DESs in the efficient extraction of BCs with anti-inflammatory properties. ChCl stands out as a highly versatile HBA due to its polarity, biodegradability, low toxicity, and strong hydrogen-bonding capabilities with various HBDs. Binary and ternary DES formulations containing ChCl, combined with sugars, polyalcohols, organic acids, or water, have been extensively studied and applied in conventional and assisted extraction methods such as ultrasound- and microwave-assisted techniques.

Phenolic compounds, particularly polyphenols and flavonoids, dominate the classes of bioactives successfully extracted using ChCl-based DESs, with curcumin, rosmarinic acid, and rutin among the frequently studied molecules. Extraction yields vary widely depending on the specific DES composition, extraction parameters, and the nature of the plant matrix, underscoring the critical influence of both HBA and HBD selection on efficiency. While some systems achieve high extraction performance, others show lower yields compared to conventional solvents, indicating that the optimization of DES formulations remains essential.

At the same time, NADESs, which combine naturally occurring HBAs and HBDs such as betaine, L-proline, amino acids, sugars, and organic acids, emerge as a promising and more sustainable alternative. These systems enable the modulation of polarity, viscosity, and extraction capacity according to the target compound. Recent studies have shown remarkable results, often surpassing conventional solvents.

Despite their advantages in the sustainable extraction of BCs, DESs face challenges such as limited recyclability, high costs, viscosity, and the need to evaluate toxicity and biodegradability for use in the pharmaceutical and food industries. Future research should focus on developing efficient recovery methods and more economical, low-viscosity formulations, as well as scaling up industrial processes through assisted technologies. Furthermore, advancing the customized design of DESs would improve their selectivity and sustainability, establishing them as a viable option for the large-scale production of natural anti-inflammatory agents and other BCs.

## Figures and Tables

**Table 2:** Extraction of bioactive compounds using DESs formed from HBAs other than ChCl–compound families, species, methods, and yields.

Bioactive Molecule	Type of Bioactive Molecule	Specie	DES		Extraction Method	Extraction (Yield (mg/g))	Reference
			Components	Ratio			
Geraniin, corilagin, ellagic acid, gallic acid	Ellaginannis	Rambutan (*Nephelium lappaceum* L.) peel	Betaine–1,2-propanediol	1–2	Liquid–liquid extraction	54.29	[81]
p-coumaric acid, caffeic acid, chlorogenic acid, 4-o-caffeylquinic acid, gallic acid, protocatechuic acid, gentisic acid, vanillic acid	Phenolic acids and flavonoids	*Ribes nigrum* L. Leaf	L-proline–Propileneglycol–Water	1–2–30% Water *v*/*v*	Ultra-turrax extraction (UTE), (different minutes)	10 min– 169.5 mg GAE/g and 7.49 mg QE/g	[82]
			L-proline–Propileneglycol–Water	1–1–50% Water *v*/*v*		10 min– 221.9 mg GAE/g and 7.64 mg QE/g	
			L-proline–Propileneglycol–Water	1–1–40% Water *v*/*v*		5 min– 218 mg GAE/g and 7.79 mg QE/g	
			L-proline–Propileneglycol–Water	2–3–30% Water *v*/*v*		5 min– 239 mg GAE/g and 9.10 mg QE/g	
			L-proline– Lactic acid–Water	1–2–50% Water *v*/*v*		5 min– 74.4 mg GAE/g and 2.90 mg QE/g	
			L-proline–Lactic acid–Water	1–2–50% Water *v*/*v*		10 min– 218 mg GAE/g and 6.92 mg QE/g	
			L-proline–Lactic acid–Water	1–2–30% Water *v*/*v*		7.5 min– 256 mg GAE/g and 6.93 mg QE/g	
			L-proline–Glucose–Water	1–2–30% Water *v*/*v*		5 min– 227 mg GAE/g and 6.89 mg QE/g	
			L-proline–Glucose–Water	1–1–50% Water *v*/*v*		5 min– 192 mg GAE/g and 7.40 mg QE/g	
			L-proline–Glucose–Water	1–1–30% Water *v*/*v*		10 min– 255 mg GAE/g and 7.73 mg QE/g	
			L-proline–Propileneglycol–Water	1–2–50% Water *v*/*v*	UAE, (different minutes)	5 min– 205 mg GAE/g and 8.38 mg QE/g	
			L-proline–Propileneglycol–Water	1–1–30% Water *v*/*v*		10 min– 224 mg GAE/g and 8.66 mg QE/g	
			L-proline–Lactic acid–Water	1–2–30% Water *v*/*v*		5 min– 456 mg GAE/g and 14.3 mg QE/g	
			L-proline–Lactic acid–Water	1–1–50% Water *v*/*v*		5 min– 408.0 mg GAE/g and 12.9 mg QE/g	
			L-proline–Lactic acid–Water	1–2–30% Water *v*/*v*		10 min– 415 mg GAE/g and 10.9 mg QE/g	
			L-proline–Lactic acid–Water	1–1–50% Water *v*/*v*		10 min– 365 mg GAE/g and 10.9 mg QE/g	
			L-proline–Glucose–Water	1–1–30% Water *v*/*v*		5 min– 425 mg GAE/g and 14.6 mg QE/g	
			L-proline–Glucose–Water	1–2–30% Water *v*/*v*		10 min– 319 mg GAE/g and 11.6 mg QE/g	
Chlorophylls, carotenoids and Phycocyanin	Biological molecules	*Arthrospira platensis* (Spirulina)	Betaine–Glycerol	1–2	Freeze dried biomasses using UAE	Chlorophyll– 0.01 mg/g, carotenoid– 0 mg/g, phycocyanin– 0.2 mg/g	[83]
			Betaine–Glycerol	1–4		-	
			Betaine–Glycerol	1–8		Chlorophyll– 0.02 mg/g, carotenoid– 0.01 mg/g, phycocyanin– 0.2 mg/g	
			Glucose–Glycerol	1–2		-	
			Glucose–Glycerol	1–3		-	
			Glucose–Glycerol	1–4		-	
			Glucose–Glycerol	1–5		-	
			Glucose–Glycerol–Water	1–2–2		-	
			Glucose–Glycerol–Water	1–2–4		Chlorophyll– 0.1 mg/g, carotenoid– 0.02 mg/g, phycocyanin– 1.2 mg/g	
			Glucose–Glycerol–Betaine	1–2–4		-	
			Lactic acid–Betaine	2–1		-	
			Lactic acid–Glycerol	1–1		-	
			Menthol–Lactic acid	1–2		-	
			Menthol–Levulinic acid	1–2		Chlorophyll– 0.1 mg/g, carotenoid– 0.02 mg/g, phycocyanin– nd	
			Menthol–Octanoic acid	1–1		-	
			Octanoic acid–Lauric acid	3–1		Chlorophyll 0.1 mg/g, carotenoid– 0.03 mg/g, phycocyanin– nd	
			Nonanoic acid–Lauric acid	3–1		-	
			Nonanoic acid–Decanoic acid– Lauric acid	3–2–1		Chlorophyll– 0.1 mg/g, carotenoide– 0.02 mg/g, phycocyanin– nd	
Phenolic compounds	Polyphenols	*Mentha pulegium* (*Lamiaceae*)	Lactic acid–Glucose	5–1	UAE	164	[84]
			Lactic acid–Sodium acetate	3–1		173	
			Lactic acid–Glycine	3–1		165	
Total flavonoids	Flavonoids	*Amomum villosum* Lour. (*A. villosum*)	Betaine–Propylenglycol	1–1	UAE	65.1	[85]
			Betaine–Glycerol	1–4		66.2	
			Betaine–Urea	1–2		60.2	
			Betaine–Lactic acid	1–1		56.0	
			Betaine–Malic acid	1–2		58.7	
			Betaine–Citric acid	1–1		50.6	
			Betaine–Glucose	1–1		40.6	
			Betaine–Fructose	1–1		59.1	
			Betaine–Sorbitol	1–2		58.7	
			Betaine–Xylitol	1–1		53.0	
			Proline–Propylenglycol	1–4		58.8	
			Proline–Glycerol	1–2		54.6	
			Proline–Urea	1–1		46.1	
			Proline–Lactic acid	1–1		45.8	
			Proline–Malic acid	1–1		51.1	
			Proline–Citric acid	1–1		49.6	
			Proline–Glucose	1–1		39.0	
			Proline–Fructose	1–1		60.8	
			Proline–Sorbitol	1–2		46.4	
			Proline–Xylitol	1–1		61.1	
Quercetin	Flavonoide	*Flos sophorae*	L-proline–Glycerol–Water	2–5–10% Water *v*/*v*	UAE (room temp., 45 min)	100	[86]
Isorhamnetin	Flavonoide	*Flos sophorae*	L-proline–Glycerol–Water	2–510% Water *v*/*v*	UAE (room temp., 45 min)	12.0	
Phenolic acid	Phenolic compounds	*Olea europaea* (Virgin olive oil)	Lactic acid–Glucose–Water	6–1–6	Agitation + centrifugation + filtration	Not given	[87]
Artemisin	Lactona	*Artemisa annua*	Methyl trioctyl ammonium chloride–1-butanol	1–4	UAE (temp. 45 °C, 70 min)	7.99	[88]
Hydroxisafflor, cartomin, carthamin	Polyphenols	*Carthamus tinctorius* L. (Safflower)	Lactic acid–Glucose–Water	1–1–25% Water *v*/*v*	Heat- and stirring-assisted solid–liquid extraction	N/A in mg/g (only relative extraction yield per area–2244)	[80]
			Proline–Malic acid–Water	1–1–25% Water *v*/*v*		N/A in mg/g (only relative extraction yield per area–2813)	
			Sucrose–ChCl–Water	5–5–25% Water *v*/*v*		N/A in mg/g (only relative extraction yield per area–2680)	
cartomin			Lacticacid–Glucose–Water	1–1–25% Water *v*/*v*		N/A in mg/g (only relative extraction yield per area–2229)	
			Proline–Malic acid–Water	1–1–25% Water *v*/*v*		N/A in mg/g (only relative extraction yield per area–2925)	
			Sucrose–ChCl–Water	5–5–25% Water *v*/*v*		N/A in mg/g (only relative extraction yield per area–2591)	
Carthamin			Lactic acid–Glucose–Water	1–1–25% Water *v*/*v*		N/A in mg/g (only relative extraction yield per area–235)	
			Proline–Malic acid–Water	1–1–25% Water *v*/*v*		N/A in mg/g (only relative extraction yield per area–134)	
			Sucrose–ChCl–Water	5:5:25% Water *v*/*v*		N/A in mg/g (only relative extraction yield per area:152)	

## Data Availability

The supporting data used in this manuscript has been obtained by web of science.
